# Correlation between Lower Esophageal Sphincter Metrics on High-Resolution Manometry and the Clinical Presentation of Patients with Newly Diagnosed Achalasia

**DOI:** 10.3390/diagnostics13061136

**Published:** 2023-03-16

**Authors:** Daniel L. Cohen, Eyal Avivi, Anton Bermont, Fahmi Shibli, Narges Azzam, Basem Hijazi, Fadi Abu Baker, Vered Richter, Haim Shirin, Amir Mari

**Affiliations:** 1 The Gonczarowski Family Institute of Gastroenterology and Liver Diseases, Shamir (Assaf Harofeh) Medical Center, Zerifin 703000, Israel; 2 Gastroenterology and Hepatology Institute, HaEmek Medical Center, Afula 1834111, Israel; 3 Gastroenterology and Endoscopy Unit, Nazareth EMMS Hospital, Nazareth 16100, Israel; 4 Faculty of Medicine, Bar Ilan University, Safed 1311502, Israel; 5 Gastroenterology and Hepatology Institute, Hillel Yaffe Medical Center, Hadera 38100, Israel; 6 Sackler School of Medicine, Tel Aviv University, Tel Aviv 69978, Israel

**Keywords:** achalasia, manometry, signs and symptoms, esophageal motility disorders, endoscopy

## Abstract

Background: Achalasia is characterized by aperistalsis with poor relaxation of the lower esophageal sphincter (LES). We aimed to systematically assess whether LES metrics on high-resolution manometry (HRM) correlate with the symptomatic or endoscopic presentation of patients with achalasia. Methods: A retrospective study was performed at two tertiary medical centers. All cases of newly diagnosed, untreated achalasia were reviewed for demographics, symptoms, and endoscopic findings. These were correlated with HRM metrics, including LES basal pressure (LESP), integrated relaxation pressure (IRP), percent LES relaxation, and esophagogastric junction (EGJ) morphology. Results: 108 achalasia patients were included; 56 (51.9%) were men, with a mean overall age of 55.6 ± 17.9 years old. Achalasia subtypes included 23.1% with Type I, 65.7% Type II, and 11.1% Type III. Mean LESP was 40.9 ± 13.7 mmHg, IRP 26.8 ± 11.5 mmHg, with 36% ± 20% LES relaxation. On univariate analyses, a higher IRP was associated with age < 50 (*p* = 0.028), female sex (*p* = 0.030), Arab ethnicity (*p* < 0.001), weight loss (*p* = 0.016), a tortuous esophagus (*p* = 0.036), and resistance at the EGJ (*p* = 0.033). However, on multivariate regression analyses, only ethnicity remained significantly associated with IRP. No unique variables were associated with either LESP or percent LES relaxation. Achalasia subtype and Eckardt score were not associated with any LES metrics. Non-Type 1 EGJ morphology was associated with a lower LESP. Conclusions: LES metrics on HRM do not appear to correlate with the clinical or endoscopic presentation of patients with untreated achalasia.

## 1. Introduction

Achalasia is a rare neurological disorder characterized by the absence of esophageal peristalsis and the inability of the lower esophageal sphincter (LES) to properly relax [1]. It typically presents with symptoms of dysphagia, chest pain, vomiting, and weight loss [1,2,3]. Achalasia is a chronic lifetime condition that profoundly disturbs patients’ quality of life and work productivity [1].

High-resolution manometry (HRM) is the gold standard for evaluating esophageal motility and LES function [1]. HRM studies are performed and interpreted according to the Chicago classification, currently in its fourth version (CCv4.0) [4]. Per the Chicago classification, achalasia is defined as an abnormal median integrated relaxation pressure (IRP) value with 100% failed peristalsis. IRP is a measure of the deglutitive relaxation of the LES. It is calculated as the mean of the maximal deglutitive relaxation in the 4-second window beginning at UES relaxation. Achalasia is further divided into three subtypes: Type I (with absent contractility), Type II (with panesophageal pressurization), and Type III (with spasm) [4].

While manometric measurements of the LES, specifically IRP, are required to diagnose achalasia, few studies have evaluated the correlation between LES metrics and clinical presentations. One study from the era of conventional line-tracing manometry showed that higher LES relaxation pressures were associated with higher total symptom scores and, specifically, regurgitation [5]. However, a more recent study showed that there was no correlation between IRP or basal LES pressure and symptoms when using the overall Ekhardt score or its dysphagia component [6]. Additionally, no studies have evaluated the relationship between LES metrics and the typical endoscopic findings in achalasia.

Further, while IRP is the metric used for the diagnosis of achalasia, no other LES metrics are incorporated into the diagnostic criteria of the Chicago classification [4]. Hence, other evaluations of the LES, including LES basal pressure, percent LES relaxation, and esophagogastric junction (EGJ) morphology, have been rarely described in respect of achalasia [7].

Therefore, we aim to evaluate if there are correlations between LES metrics on HRM and the clinical presentation of newly diagnosed achalasia patients, including both symptomatology and endoscopic findings. We hypothesize that higher LES pressures may suggest a more significantly closed EGJ and, thus, more significant symptoms.

## 2. Materials and Methods

### 2.1. Study Design

A retrospective study was performed at two tertiary medical centers. HRM studies were reviewed for all patients newly diagnosed with achalasia from 2018 to 2022. Demographic data, including age, gender, ethnicity, body mass index (BMI), and comorbidities were recorded. Presenting symptoms, including dysphagia, chest pain, and weight loss, were taken from the patients’ charts. Due to linguistic difficulties in distinguishing between certain terms, as well as the setup of the electronic medical records, it was frequently impossible to differentiate between regurgitation, vomiting, heartburn, and reflux symptoms. Thus, these symptoms were combined into a single variable and considered positive if any one of these symptoms was present. Eckardt scores were used when available [8].

Subjects under the age of 18 were excluded. If a subject underwent multiple studies during the study period, only the first (index) study was included. Patients with previously diagnosed or treated achalasia were excluded. All subjects required a complete HRM study for inclusion. Any incomplete HRM study was excluded, for example, any study with less than eight evaluable swallows, failure to traverse the LES, or any technical difficulties.

### 2.2. HRM Protocol and Interpretation

At each center, HRM was performed using the same system (ManoScanAR, Medtronic, Minneapolis, MN, USA). HRM studies were performed according to the standard protocol, as per the Chicago classification [4], and the results were recorded. The following parameters were evaluated: number of swallows completed, IRP, LES basal pressure, EGJ morphology, and the subtype of achalasia-I, -II, or -III. As few patients had Type 2 or Type 3 EGJ morphology, these subjects were combined together for statistical analysis. Additionally, the percentage of LES relaxation was calculated using the LES basal pressure as the baseline and the IRP as the post-swallow relaxation pressure.

All HRM studies were reviewed by an expert in esophageal motility (D.L.C. or A.M.) to confirm the diagnosis of achalasia and its subtype. Additionally, the HRM metrics used for this study were obtained from this re-analysis and not simply taken from the original report.

### 2.3. Endoscopic Findings at Gastroscopy

All patients’ endoscopy reports were reviewed by senior gastroenterologists for suggestive findings of achalasia, including: the presence of esophageal residue, a dilated esophagus, a tortuous esophagus, and resistance at the EGJ [9]. Residue was defined as the presence of either residual liquid or solid material in the esophagus. A dilated esophagus was defined solely based on the endoscopist’s description and did not include any radiographic studies. If any of these endoscopic findings were not reported in the procedure report, then it was recorded as negative. If endoscopy procedure images were available, these were evaluated secondarily to confirm what was written in the endoscopy report text.

### 2.4. Statistical Analysis

Categorical variables are presented as frequency and percentage. All continuous variables were normally distributed and are presented as mean and standard deviation. The Pearson chi-square test and Fisher’s exact test were used to compare the categorical variables. The Mann–Whitney test or Student t-test was performed to compare continuous variables. Multiple linear regression models (enter and stepwise methods) were performed to assess the effects of the independent variables on IRP. All statistical tests were two-sided, with a *p*-value of <0.05 considered significant. Analyses was performed using IBM SPSS Statistics v.28 software.

## 3. Results

### 3.1. Details of the Achalasia Cohort

The cohort consisted of 108 newly diagnosed achalasia patients. Details of the cohort—including demographic, medical, symptomatic, endoscopic, and manometric data—can be found in Table 1.

The mean age was 55.6 ± 17.9 years old, with 51.9% being male. Dysphagia was present in 93.5%, while chest pain, regurgitation, and weight loss were less common. For the 51 patients with an Eckardt score available, the mean score was 6.1 ± 2.3. Nearly half of the cohort were reported to have a dilated esophagus or resistance at the EGJ. The majority of cases were Type II achalasia, and over 90% had a Type 1 EGJ morphology. The mean LESP was 40.9 ± 13.7 mmHg, with an IRP of 26.8 ± 11.5 mmHg.

### 3.2. Relationship between LES Metrics and Clinical Presentation

Statistical analyses were performed to evaluate for any correlations between the LES metrics on HRM—LESP, IRP, and percent relaxation—and the clinical presentation or endoscopic findings (Table 2). In terms of demographics, higher IRP values were found in those younger than 50 years old compared to those above 50 (29.6 ± 11.4 vs. 25.2 ± 11.3, *p* = 0.028), women compared to men (29.0 ± 12.9 vs. 24.8 ± 9.7, *p* = 0.030), and in Arabs compared to Jews (32.4 ± 13.4 vs. 23.1 ± 8.3, *p* < 0.001).

Differences were also found in clinical presentations. Patients with weight loss were found to have higher LESP (43.5 ± 15.3 vs. 38.8 ± 11.8, *p* = 0.037) and IRP values (29.5 ± 12.3 vs. 24.7 ± 10.4, *p* = 0.016) compared to those without. No differences were noted in LES metrics in the other symptoms, symptom duration, or Eckardt score (for those for whom an Eckardt score was available).

Endoscopically, both a tortuous esophagus (32.1 ± 8.9 vs. 26.0 ± 11.6, *p* = 0.036) and resistance at the EGJ (29.0 ± 12.9 vs. 24.9 ± 9.7, *p* = 0.033) were associated with higher IRP values. The other endoscopic findings were not. Additionally, there were no significant differences in LES metrics among the achalasia subtypes.

In total, six demographic and clinical variables were associated with IRP (Table 2). There were no variables associated with LESP or percent LES relaxation that did not also include IRP.

### 3.3. Multivariate Regression Analyses

A multivariate regression analysis was performed, including the three clinical variables (weight loss, a tortuous esophagus, and resistance at the EGJ) found to be significantly associated with an elevated IRP. In this model, controlling for age, sex, and ethnicity, all three of the clinical variables ceased to be significantly influential on IRP (Table 3). Only Arab ethnicity remained significant. An additional regression analysis using the stepwise method also only found ethnicity to be significant (B = −9.283, *p* < 0.001, CI −13.453, −5.113).

### 3.4. Effect of EGJ Morphology on LES Metrics

Finally, the LES metrics were compared between patients with Type 1 EGJ morphology and those with either Type 2 or 3 (Table 4). These analyses found that LESP was higher in those with Type 1 morphology than the other types (41.9 ± 13.7 vs. 30.9 ± 9.5, *p* = 0.010), while IRP and percent relaxation were not affected by EGJ morphology.

## 4. Discussion

This is the first study to systematically evaluate the correlation between LES metrics on HRM and the clinical presentation of patients with achalasia. We found that although several clinical and endoscopic variables were associated with a higher IRP on univariate analysis, none of these remained significant after controlling for demographic variables.

Patients presenting with achalasia often suffer from dysphagia (90%), heartburn (70%), regurgitation or vomiting (45%), chest pain (25%), and weight loss (10%) [1]. The treatments for achalasia, which have been shown to improve symptoms, aim to open up the LES and, thus, allow food to more easily pass into the stomach. Based on this, it appears that it is the closed, unrelaxed LES that is the main cause of symptoms. Therefore, we hypothesized that HRM metrics that suggest a more tightly closed LES, such as elevated LESP or IRP, may be associated with more severe symptoms.

Since we found that no symptom or endoscopic variable was associated with LESP or percent LES relaxation alone but only in association with IRP, we focused our evaluations solely on IRP. While a higher IRP was associated with weight loss, a tortuous esophagus, and resistance at the EGJ on univariate analyses, as may be expected from poorer relaxation of the LES, these relationships did not remain significant when controlling for demographic variables. Thus, we failed to show any significant correlation between LES metrics and clinical presentation, including the Eckardt score.

The reason why increasing IRP values do not seem to cause more symptoms or more severe symptoms is unclear. It seems to support the idea that there is a threshold value at which symptoms occur when it is crossed (for example, IRP > 15 mmHg, as per the Chicago classification [4]), but that the absolute IRP value is less important. This also appears to be the case for post-treatment achalasia patients, where studies have shown that if the IRP is brought down below a certain threshold (for example, IRP < 10 mmHg after pneumatic dilation), patients clinically do well [10,11].

Our findings are similar to what has been reported in the literature. For example, Mikaeli et al. did not find a correlation between LESP and chest pain in achalasia patients [12]. More recently, Jain et al. evaluated the correlation between IRP and both the total Eckardt score and the individual dysphagia component in achalasia patients [6]. They found that while there was a univariate correlation between IRP and both of these scores, both became non-significant in multivariable logistic regression analyses. In addition to IRP, they evaluated the correlation between the distensibility index (DI) measured by functional luminal imaging probe (FLIP) and symptoms, finding that DI alone correlated with dysphagia score. Thus, they concluded that DI appears to be a better predictor of symptoms in achalasia than HRM metrics.

Demographics are known to play a role in IRP. Similar to our findings, male gender and older age have been previously reported to be associated with lower IRP values in achalasia patients [13,14]. Surprisingly, our study found ethnicity to be even more significantly associated with IRP values. The reason for this is unclear, but it may be related to genetic variations in immunogenic HLA haplotypes that have been reported in achalasia populations [15,16]. Future studies evaluating the role of ethnicity and genetics in achalasia patients are certainly warranted [17].

We also found that IRP did not predict the subtype of achalasia, although Type III achalasia trended towards a higher IRP. Other studies have also evaluated the relationship between IRP and achalasia subtypes with conflicting results. Blais et al. found no differences in IRP between the subtypes of achalasia and concluded that IRP cannot be used to differentiate between the three subtypes of achalasia [18]. However, other studies have shown higher IRP measurements in Types II and III compared to Type I achalasia [19,20].

Associations between EGJ morphology and HRM metrics have previously been assessed, mainly in patients with gastroesophageal reflux disease (GERD). The presence of Type 2 or 3 EGJ morphology, which manometrically signifies the presence of a hiatal hernia, has been independently associated with pathologic reflux [21,22,23]. Further, Type 3 morphology has been associated with reduced LESP [21]. In our study, we found that the presence of Type 2 or 3 EGJ morphology in achalasia patients was associated with a lower LESP than Type 1 morphology, but EGJ morphology did not impact IRP values.

Our study has some limitations. As this was a retrospective study, data on the clinical presentation of patients and endoscopic findings were collected via chart review. As such, details of their symptoms and endoscopic findings may not have been thoroughly reported in their chart and, therefore, may be lacking. Additionally, while data on the presence of symptoms was collected for all subjects, an Eckardt score was only available for about half the subjects. Finally, data on complementary studies such as barium esophogograms were not available, nor were there data on how they were treated for achalasia or their clinical course.

In conclusion, our findings help better understand the function of the LES in achalasia patients, although LES metrics on HRM do not appear to correlate with the clinical or endoscopic presentation of patients with newly diagnosed achalasia.

## Figures and Tables

**Table 1:** Demographics, clinical presentation, and manometric variables of the achalasia cohort.

Variable	Result (*n* = 108)
Demographics	
Age	55.6 +/− 17.9
Sex	
Male	56 (51.9%)
Female	52 (48.1%)
Ethnicity	
Arab	43 (39.8%)
Jewish	65 (60.2%)
Medical history	
BMI	26.4 +/− 7.2
Smoking	36 (33.3%)
Stroke	8 (7.4%)
Ischemic heart disease	20 (18.5%)
Diabetes	25 (23.1%)
Symptoms	
Dysphagia	101 (93.5%)
Chest pain	38 (35.2%)
Regurgitation/reflux	63 (58.3%)
Weight loss	48 (44.4%)
Eckardt score	6.1 +/− 2.3
Symptom duration	
<1 year	40 (37.0%)
1–3 years	24 (22.2%)
>3 years	44 (40.7%)
Endoscopic findings	
Residue in esophagus	41 (38.0%)
Dilated esophagus	51 (47.2%)
Tortuous esophagus	13 (12.0%)
Resistance at EGJ	50 (46.3%)
Manometry findings	
Achalasia subtype	
Type I	25 (23.1%)
Type II	71 (65.7%)
Type III	12 (11.1%)
EGJ morphology	
Type 1	99 (91.7%)
Type 2 or 3	9 (8.3%)
LESP	40.9 +/− 13.7
IRP	26.8 +/− 11.5
% LES relaxation	36 +/− 20

BMI: body mass index; EGJ: esophagogastric junction; LESP: lower esophageal sphincter basal pressure; IRP: integrated relaxation pressure; LES lower esophageal sphincter.

**Table 2:** Correlation between LES metrics and clinical presentation.

Variable		LESP	*p*-Value	IRP	*p*-Value	% Relaxation	*p*-Value
**Demographics**							
Age	<50	42.8 +/− 13.7	0.140	29.6 +/− 11.4	**0.028**	34 +/− 21	0.167
	≥50	39.8 +/− 13.6		25.2 +/− 11.3		38 +/− 19	
Sex	Male	39.0 +/− 12.4	0.068	24.8 +/− 9.7	**0.030**	38 +/− 20	0.129
	Female	42.9 +/− 14.7		29.0 +/− 12.9		34 +/− 20	
Ethnicity	Arab	41.0 +/− 13.0	0.413	32.4 +/− 13.4	**<0.001**	31 +/− 16	**0.022**
	Jewish	40.7 +/− 14.1		23.1 +/− 8.3		39 +/− 22	
**Symptoms**							
Dysphagia	yes	40.9 +/− 13.9	0.486	27.2 +/− 11.7	0.080	36 +/− 20	0.34
	no	41.1 +/− 10.0		20.9 +/− 4.9		33 +/− 19	
Chest pain	yes	38.8 +/− 11.3	0.124	26.7 +/− 12.9	0.476	35 +/− 20	0.352
	no	42.0 +/− 14.7		26.8 +/− 10.7		37 +/− 20	
Regurgitation/reflux	yes	42.1 +/− 14.5	0.142	27.5 +/− 11.9	0.224	35 +/− 18	0.251
	no	39.2 +/− 12.2		25.8 +/− 10.8		38 +/− 22	
Weight loss	yes	43.5 +/− 15.3	**0.037**	29.5 +/− 12.3	**0.016**	34 +/− 20	0.192
	no	38.8 +/− 11.8		24.7 +/− 10.4		38 +/− 20	
Eckardt score							
	0 to 4	39.5 +/− 14.5	0.991	33.5 +/− 11.1	0.335	30 +/− 11	0.700
	5 to 7	44.1 +/− 12.1		31.7 +/− 12.7		32 +/− 16	
	8 to 12	38.6 +/− 10.3		31.1 +/− 14.2		35 +/− 18	
Symptom duration							
	<1 year	41.8 +/− 12.5	0.829	26.6 +/− 12.6	0.146	41 +/− 20	0.357
	1–3 years	43.4 +/− 14.4		25.7 +/− 8.5		33 +/− 20	
	>3 years	38.7 +/− 14.2		27.5 +/− 12.0		33 +/− 20	
**Endoscopic findings**							
Residue in esophagus	yes	38.8 +/− 14.3	0.111	25.8 +/− 11.3	0.248	33 +/− 22	0.103
	no	42.2 +/− 13.1		27.4 +/− 11.6		38 +/− 19	
Dilated esophagus	yes	40.5 +/− 13.1	0.391	27.3 +/− 10.8	0.325	35 +/− 20	0.281
	no	41.2 +/− 14.2		26.3 +/− 12.1		37 +/− 20	
Tortuous esophagus	yes	34.6 +/− 11.8	**0.039**	32.1 +/− 8.9	**0.036**	33 +/− 18	0.298
	no	41.7 +/− 13.7		26.0 +/− 11.6		36 +/− 20	
Resistance at EGJ	yes	40.1 +/− 13.6	0.292	29.0 +/− 12.9	**0.033**	36 +/− 22	0.404
	no	41.6 +/− 13.7		24.9 +/− 9.7		37 +/− 18	
**Manometry findings**							
Achalasia subtype							
Type I	yes	39.1 +/− 14.8	0.232	25.6 +/− 9.7	0.275	35 +/− 19	0.344
	no	41.4 +/− 13.3		27.1 +/− 12.0		37 +/− 20	
Type II	yes	41.7 +/− 12.7	0.186	26.8 +/− 10.9	0.484	37 +/− 21	0.149
	no	39.2 +/− 15.5		26.7 +/− 12.7		33 +/− 18	
Type III	yes	37.8 +/− 17.6	0.205	31.4 +/− 17.2	0.070	29 +/− 16	0.082
	no	41.3 +/− 13.1		26.2 +/− 10.5		37 +/− 20	

EGJ: esophagogastric junction; LESP: lower esophageal sphincter basal pressure; IRP: integrated relaxation pressure. *p*-values in bold represent statistically significant findings.

**Table 3:** Multivariate regression analysis.

Variable	*t* Score	*p*-Value	95% Confidence Interval
Age	−0.500	0.618	−0.144, 0.086
Sex	1.934	0.056	−0.101, 7.992
Ethnicity	−3.396	**<0.001**	−12.117, −3.180
Weight loss	1.513	0.134	−0.995, 7.385
Tortuous esophagus	0.577	0.565	−4.672, 8.507
Resistance at EGJ	1.709	0.091	−0.570, 7.651

EGJ: esophagogastric junction. *p*-values in bold represent statistically significant findings.

**Table 4:** Correlation between EGJ morphology and LES metrics.

	Type 1	Type 2 or 3	*p*-Value
LESP	41.9 +/− 13.7	30.9 +/− 9.5	**0.010**
IRP	26.7 +/− 11.5	27.8 +/− 12.0	0.389
% relaxation	37 +/− 19	29 +/− 26	0.138

EGJ: esophagogastric junction; LES: lower esophageal sphincter pressure; LESP: lower esophageal sphincter basal pressure; IRP: integrated relaxation pressure. *p*-values in bold represent statistically significant findings.

## Data Availability

The database is available from the corresponding author upon reasonable request.
